# Gender-Specific Correlates of Suicidal Behaviour: Insights from the Interpersonal Theory of Suicide

**DOI:** 10.3390/jcm15041335

**Published:** 2026-02-08

**Authors:** Anna Lubas-Grzyb, Danuta Rode, Magdalena Rode, Alison J. Marganski

**Affiliations:** 1 Clinical Psychiatric Hospital in Rybnik, 44-200 Rybnik, Poland; alpsycholog@gmail.com; 2 Faculty of Psychology in Katowice, SWPS University, 03-815 Warsaw, Poland; drode@swps.edu.pl; 3 Institute of Psychology, Faculty of Social Sciences, University of Silesia, 40-007 Katowice, Poland; 4 Department of Anthropology, Criminology, and Sociology, Le Moyne College, Syracuse, NY 13214, USA; marganaj@lemoyne.edu

**Keywords:** suicide, suicide attempt, suicidal behavior, gender, interpersonal theory of suicide, personality, hopelessness

## Abstract

**Background/Objectives:** This study examined gender-specific psychological and interpersonal correlates of suicidal behaviour using the framework of the Interpersonal Theory of Suicide (IPTS). **Methods**: The study included a total of 181 respondents from a clinical group (N = 93) and a control group (N = 88). Logistic regression analyses were conducted separately for women (N = 86) and men (N = 80) for cases that met leverage values (LEV) ≤ 0.2. Variables included personality traits, coping style, hopelessness, self-esteem, hope, perceived burdensomeness, thwarted belongingness, and acquired capacity for suicide. Interaction terms were also tested. **Results**: Among women, hopelessness (Exp(B) = 1.37; *p* = 0.038) and perceived burdensomeness (Exp(B) = 1.12; *p* = 0.033) were identified as significant correlates of suicidal behaviour. Among men, an avoidance-focused style (Exp(B) = 1.18; *p* = 0.009) and the interaction of general capacity for suicide x perceived burdensomeness x thwarted belongingness (Exp(B) = 5.29; *p* = 0.043) emerged as significant correlates. Further analysis indicated that thwarted belongingness became a significant factor in men only when perceived burdensomeness and capacity for suicide were high (Nagelkerke R^2^ = 0.33; Exp(B) = 1.17; *p* = 0.042). **Conclusions**: Gendered expressions of suicidality appear to follow distinct pathways. Within the IPTS framework, women’s suicidality is more closely shaped by internalized cognitive and affective processes, including hopelessness and perceived burdensomeness, whereas men’s behaviour is influenced by maladaptive coping, social disconnection, and acquired capacity for suicide. These findings highlight the importance of gender-specific prevention and intervention strategies across clinical and community contexts. Early identification of these correlates may reduce suicidal intent, prevent rehospitalization, and improve mental health outcomes.

## 1. Introduction

Suicide is a leading cause of death among people aged 15–29 [1]. It is defined as deliberate, self-inflicted behaviour of an individual directed against themselves, resulting in their death [2], and is one of the most devastating manifestations of suicidal ideation. Suicidal behaviour is characterized by an individual (intrapsychic) approach to a mental health crisis stemming from a breakdown in the ability to deal with stress. The latest diagnostic criteria for mental disorders (DSM-5-TR) classifies suicidal behaviour disorder (SBD) as a condition placed under “Other Conditions That May Be a Focus of Clinical Attention” for individuals who have attempted to end their own lives at least one time within the last 24 months. Research suggests that men die by suicide significantly more often than women [2,3,4,5], and this holds true globally [1]. However, focusing solely on suicide mortality overlooks many individuals who are at risk for, or engage in, suicidal behaviours. Studies indicate that women experience suicidal ideation, engage in suicidal behaviour, and attempt suicide at rates similar to or even higher than those of men [6]. One of the most frequently cited explanations for this gender disparity concerns the method employed in self-harm: men are more likely to use highly lethal means, such as firearms or hanging, whereas women more often resort to less immediately fatal methods, such as poisoning or medication overdose. Given the higher lethality of firearms compared to overdoses, this difference in method selection translates to men’s higher suicide mortality rates [7,8,9,10]. Nonetheless, other factors may also shape gender differences in suicidality [11] and warrant further consideration.

Suicidal behaviour arises from a complex interplay of many factors (e.g., biological, psychological, sociological, cultural, and environmental). Gender differences are evident not only in the methods used, but also in the underlying factors influencing decisions to engage in suicidal behaviour. These differences are shaped by cultural patterns as well as intrapsychic and biological variables. Consequently, variations between men and women may exist in certain psychological processes or internal cognitive–affective mechanisms (e.g., thought patterns) that influence suicidal actions, which carries implications for the development of gender-responsive intervention/prevention strategies that hold the potential to save lives.

Research on men who exhibit suicidal behaviours indicates that high levels of emotional distress and ongoing psychosocial challenges adversely affect their functioning. Men have been found to be less likely than women to seek help from mental health professionals or access support during a mental health crisis, which reflects a pattern often linked to dominant or hegemonic masculinity norms embedded in their social environments [12]. They are commonly characterized as having an avoidant coping style [13]. This culturally influenced reluctance to seek help increases vulnerability. For some men, suicidal behaviour and limited help-seeking are associated with stoicism, whereby emotions are suppressed and hardship is endured. For others, these behaviours may relate to sensation seeking and the pursuit of intense experiences that allows them to tolerate pain or overcome fear of death [14]. Men who have attempted suicide may struggle to recognize their level of risk, minimize or internalize their pain, and/or lack intrinsic motivation to seek assistance.

In contrast, less research has focused on women who engage in suicidal behaviours. However, existing studies suggest that traditional gender roles also contribute to heightened vulnerability. Women often experience pressure to manage multiple responsibilities (e.g., childcare, household duties, emotional labour, etc.), which can contribute to psychological distress and feelings of despair [15,16]. Those who perceive themselves as “bad mothers”, feel like failures, or experience entrapment or defeat exhibit a higher risk of suicidal behaviour [17]. Furthermore, women who feel responsible for making a relationship work are at increased risk for suicidality when there is the presence of trauma or abuse within intimate or other interpersonal relationships [18]. Social isolation, which is also a consequence of such trauma, can further increase risk. In patriarchal contexts, socialization processes that emphasize self-sacrifice, relational harmony, and emotional caretaking may undermine women’s safety and well-being. Additional life events and transitions, such as pregnancy [19] and menopause [20], have also been associated with increased vulnerability to suicidal behaviours.

Men and women differ in how they perceive themselves and interpret their experiences, which is largely shaped by cultural norms that influence cognition and, in turn, behaviour. One of the most recent theoretical frameworks for understanding suicidal behaviour is the Interpersonal Theory of Suicide (IPTS), which underlies the present research. Joiner [19] conceptualized suicidal thoughts and behaviours as existing along a continuum. According to this theory, the desire for suicide emerges from the coexistence of thwarted belongingness (TB) and perceived burdensomeness (PB), while the capability for suicide involves distinguishing between the desire to commit suicide and the capacity for suicide. The IPTS has been supported by numerous studies confirming its assumptions regarding the importance of burdensomeness, capability for suicide and thwarted belongingness in suicidal ideation and behaviour [21,22,23]. Nonetheless, the IPTS has been criticized for, among other things, its broad conceptualization of suicide, limitations in measurement reliability, and methodological flaws in meta-analyses intended to assess its validity [24]. More recent developments have extended the IPTS to include hopelessness as a construct [25], strengthening its explanatory power in assessing suicide risk. However, the theory still does not account for multisystemic factors or triggering events that may play a role in suicide among individuals without diagnosed mental disorders or prior suicidal ideation [26].

The current study applies the IPTS as a conceptual framework to examine factors associated with intentional suicidal behaviours among a group of men and a group of women in Poland. Specifically, the study aims to identify gender differences in personality variables and perceived burdensomeness, thwarted belongingness, and capability for suicide among individuals who have attempted suicide. Understanding these subjective and gender-differentiated variables may inform more individualized and gender-responsive approaches to suicide prevention and crisis intervention. The central hypothesis posits that there are significant gender differences across these psychological dimensions and their associations with suicidal behaviour.

## 2. Materials and Methods

A total of 181 participants (N = 92 female; N = 89 men) were included in this study. Among them, 93 respondents (N = 93; age: M = 32.04, *SD* = 10.29) formed the clinical group, consisting of women (N = 45) and men (N = 48) who had been hospitalized in a psychiatric facility due to a suicidal attempt. The remaining 88 participants (N = 88; age: M = 35.43, *SD* = 10.35) comprised the control group, consisting of women (N = 47) and men (N = 41) with no current or past suicidal behaviours.

Participants in the clinical group met the criteria for suicidal behaviour disorder (SBD) as outlined in the DSM-5. Inclusion criteria required that individuals had attempted suicide within the previous 24 months, did not meet the diagnostic criteria for non-suicidal self-injury (NSSI), and were free of delirium, confusion, and political/religious motives for their actions. Participants presenting with current major depressive episodes, active psychosis, or acute substance withdrawal were excluded to reduce confounding effects and ensure participant safety. These conditions are known to exert strong influences on suicidal ideation, and their inclusion could obscure relationships among the primary study variables. Excluding such cases therefore strengthened the internal validity and interpretability of findings by allowing for examination of suicidal ideation and behaviour in the absence of acute psychiatric or physiological crises. Reporting suicidal ideation was not mandatory. Table 1 includes the classification criteria for the study group.

We intentionally include individuals who had attempted suicide without diagnosed mental disorders in the study group, as such individuals remain at substantial risk for future attempts. Identifying the psychological correlates of suicidal behaviour in this population is critical for improving the prediction of suicide risk.

The clinical group consisted of 93 participants aged 18 to 56 years who met the DSM-5 criteria for suicidal behaviour disorder (DSM-5). This group included women (N = 45) and men (N = 48). Among women, 12 (26.67%) had completed higher education, 21 (46.67%) had completed secondary education, 7 (15.56%) had completed vocational education, and 5 (11.11%) had completed primary education. Among men, 8 (16.67%) had completed higher education, 25 (52.08%) had completed secondary education, 7 (14.58%) had completed vocational education, and 8 (16.67%) had completed primary education. Among women, 24 participants (53.33%) were parents, while 21 (46.67%) were childless. Among men, 19 participants (39.58%) had children, whereas 29 (60.42%) were childless. Regarding employment, 37 women (82.22%) were employed, while 8 (17.78%) were unemployed or economically inactive. Among men, 28 (58.33%) were employed, and 20 (41.67%) were unemployed or inactive. Participants exhibiting suicidal behaviours most frequently reported suicidal ideation (75.27%), while the majority had not attempted suicide in the past (56.99%).

The control group consisted of 88 participants aged 20 to 60 years, including 47 women and 41 men, all without current or past suicidal behaviours. Among women, 18 participants (38.30%) had completed higher education, 14 (29.79%) had completed secondary education, 10 (21.28%) had completed vocational education, and 5 (10.64%) had completed primary education. Among men, 16 participants (39.02%) had completed higher education, 17 (41.46%) had completed secondary education, 4 (9.76%) had completed vocational education, and 4 (9.76%) had completed primary education. Within the control group, 29 women (61.70%) were parents and 18 (38.30%) were childless, while 22 men (53.66%) were parents and 19 (46.34%) were childless. Regarding employment, 41 women (87.23%) and 33 men (80.49%) were employed, while 6 women (12.8%) and 8 men (19.51%) were economically inactive.

The study was conducted at the Hospital for the Neurologically and Mentally Ill in Rybnik (Poland; currently named Clinical Psychiatric Hospital in Rybnik) from November 2021 to December 2022. Prior to commencement, approval was obtained from the hospital director and the heads of wards. The study’s research protocol and procedures were reviewed and approved by the hospital’s Research Ethics Committee, which identified no potential breaches of ethical principles. Assessments were conducted at the hospital’s Admission Wards. Before implementation, a team of psychologists and a psychiatrist reviewed the tests to ensure they did not impose undue burden on patients. The study was deemed appropriate and feasible as originally proposed. All participants were provided with written informed consent, which was included in the patients’ medical records. The study excluded minors. The consent form outlined information about participants’ rights to anonymity, voluntariness, withdrawing at any time, minimal risk to well-being, and access to psychological care or support.

Participants in the clinical group were first screened via an initial interview and subsequently completed the study procedure, which lasted approximately two hours and was divided into two stages. In the first stage, participants completed a set of questionnaires and some psychological assessment; the second stage involved the remaining psychological tests. Seven instruments were administered: the NEO Five Factor Inventory (NEO-FFI) by Costa and McCrae [27]; the Beck Hopelessness Scale (BHS) [28]; the Coping Inventory for Stressful Situations (CISS) by Endler and Parker [29]; the Interpersonal Needs Questionnaire (INQ-15) by Van Orden et al. [30]; the Acquired Capability for Suicide Scale (ACSS-20) by Ribeiro et al. [31]; the Coopersmith Self-Esteem Inventory (CSEI) by Ryden [32]; and the Hope for Success Questionnaire by Łaguna et al. [33].

The following instruments were used:

- The NEO Five-Factor Inventory (NEO-FFI; P. T. Costa & R. R. McCrae; Polish adaptation [27] assesses personality traits. Only items pertaining to the Neuroticism scale (12 items) were used. Cronbach’s α = 0.86.
- The Beck Hopelessness Scale (BHS) [28] assesses hopelessness.
- The Coping Inventory for Stressful Situations (CISS; N. S. Endler & J. D. A. Parker; Polish adaptation [29] is a 48-item self-report measure designed for adults describing coping behaviors that individuals exhibit in stressful situations. Cronbach’s α = 0.74 to 0.88 across the three main coping dimensions.
- The Interpersonal Needs Questionnaire–15 (INQ-15) [30] assesses thwarted belongingness (TB) and perceived burdensomeness (PB) using 15 items rated on a 7-point Likert scale; cross-language correlation coefficients = 0.87–0.96, confirming strong linguistic equivalence.
- The Acquired Capability for Suicide Scale–20 (ACSS-20) [31] measures acquired capability for suicide. Cross-language correlation = 0.79–0.86, which indicates cross-language reliability.
- The Coopersmith Self-Esteem Inventory (CSEI; adult version adapted) [32] is a self-report instrument measuring global self-esteem; cross-language correlation = 0.82–0.87 (subscales not used).
- The Hope for Success Questionnaire (KNS) [33] consists of 12 items measuring pathways thinking and agency thinking. Cronbach’s α of the Polish version = 0.82 (0.76 to 0.86 across five previous studies). Reliability coefficients for the subscales were 0.72 for pathways thinking and 0.74 for agency thinking. The instrument demonstrated satisfactory temporal stability.

In the present study, analyses were restricted to the neuroticism dimension of the NEO-FFI. This decision was guided by substantial empirical evidence demonstrating that neuroticism is the personality trait most consistently and robustly associated with suicidal ideation, suicide attempts, and suicide-related behaviours across both clinical and community samples [34,35]. From a theoretical perspective, neuroticism is conceptually aligned with the Interpersonal-Psychological Theory of Suicide (IPTS) [35]. Individuals high in neuroticism are characterized by heightened emotional reactivity and negative affectivity, which may intensify experiences of perceived burdensomeness and thwarted belongingness—the two proximal psychological states proposed by IPTS to underlie suicidal desire [36,37].

Although other Big Five traits have been examined in relation to suicidality, findings for these dimensions are generally less consistent, whereas neuroticism demonstrates the strongest and most reproducible associations [34]. On this basis, neuroticism was selected as the most theoretically relevant and empirically supported personality dimension for the present analyses.

All instruments requiring translation followed established cross-cultural adaptation procedures [38], including independent parallel translation, reconciliation, pilot testing, and verification of linguistic equivalence in a bilingual sample.

The control group consisted of 88 volunteers with no history of suicide attempts, psychiatric treatment, or active psychotic symptoms. Participants were recruited online via general public social media groups (including local community and university groups and the Facebook page of a psychology clinic) and institutional websites. Recruitment employed a snowball sampling procedure whereby initial participants were invited to share the study link with other eligible individuals. Participation was voluntary and based on self-selection. Screening questions confirmed the absence of past or current suicidal thoughts, suicide attempts, or psychiatric disorders prior to study inclusion, and all participants provided informed consent.

Only variables that met two criteria were included in the analyses: (1) they were not linearly interdependent as indicated by a variance inflation factor (VIF < 5) [39] and (2) they had a theoretically supported influence on suicidal behaviours. Analyses were conducted only for cases that met the LEV ≤ 0.2 condition, ensuring the exclusion of influential outliers. After applying the criteria, the final sample consisted of 166 people, divided into two groups based on gender. Given the study design, time constraints, and the number of items in the instruments, an events per variable (EPV) value of 5 was assumed. Interaction terms between predictors were included in the regression models, which increased the total number of variables considered.

## 3. Results

Based on the theoretical framework and methodological considerations, including sample size limitations and logistic regression assumptions, the following variables were included in the analyses: avoidance-focused style, hopelessness, neuroticism, hopefulness, self-esteem, perceived burdensomeness, thwarted belongingness, and general capacity for suicide. In addition, in line with the IPTS, three interactions were included in the model: perceived burdensomeness x thwarted belongingness; general capacity for suicide x perceived burdensomeness x thwarted belongingness; and hopelessness x perceived burdensomeness x thwarted belongingness. Variables excluded from the model did not meet the criteria for linear independence (LEV ≤ 0.2) or the assumption of linearity between the continuous independent variables and the logit of the dependent variable.

Table 2 includes the results obtained using logistic regression analysis for women (N = 86), while Table 3 shows the results obtained for men (N = 80).

The logistic regression model for the women’s group demonstrated good fit according to the Hosmer–Lemeshow test (*p* > 0.05) and explained 58% of the variance in suicidal behaviour. Hopelessness (Exp(B) = 1.37; *p* = 0.038) and perceived burdensomeness (Exp(B) = 1.12; *p* = 0.033) emerged as significant correlates of suicidal behaviour, both positively associated with suicidal behaviour. Specifically, a one-unit increase in hopelessness was associated with a 37% higher likelihood of suicidal behaviour, while a one-unit increase in perceived burdensomeness was associated with a 12% higher likelihood of suicidal behaviour.

For the men’s group, the model also demonstrated good fit according to the Hosmer–Lemeshow test (*p* > 0.05) and explained 65% of the variance in suicidal behaviour. Avoidance-focused style (Exp(B) = 1.18; *p* = 0.009) and interaction between general capacity for suicide x perceived burdensomeness x thwarted belongingness (Exp(B) = 5.29; *p* = 0.043) were significant correlates of suicidal behaviours. A one-unit increase in avoidance-focused style increased the likelihood of suicidal behaviour by 18%. For three-way interaction, higher combined values of general capacity for suicide, perceived burdensomeness and thwarted belongingness were associated with a 29% increase in the likelihood of suicidal behaviours.

To further explore the interaction in the men’s group, a model with thwarted belongingness and perceived burdensomeness and four interaction combinations was tested (Table 4 and Table 5). Analysis indicated that thwarted belongingness becomes significant when both perceived burdensomeness and general capacity for suicide reach high values. Nagelkerke’s R^2^ statistic indicates this interaction accounted for 33% of the variance in suicidal behaviour (Nagelkerke’s R^2^ = 0.33; Exp(B) = 1.17, *p* = 0.042). The remaining interaction terms were not statistically significant.

## 4. Discussion

In the women’s group, hopelessness and perceived burdensomeness emerged as significant correlates of suicidal behaviour [20,40]. This finding suggests that hopelessness may operate more directly and independently than anticipated for women. Rather than being a byproduct of IPTS factors, hopelessness may serve as a central psychological state mediating the relationship between life stressors, gendered experiences, and suicidal behaviour. It may capture how women integrate social pain, chronic stress, and gendered vulnerabilities (e.g., role expectations, trauma, or relational loss) into suicidal thinking. This aligns with the notion of a gendered pathway in which women’s suicidal behaviors are more strongly shaped by internalized cognitive styles such as ruminative and future-oriented negative thinking rather than by externalized expressions of interpersonal disconnection or fearlessness. The persistent significance of perceived burdensomeness in women supports the IPTS model, while also indicating that its predictive utility may depend on the inclusion of cognitive variables like hopelessness.

Perceived burdensomeness may manifest as self-directed hostility where women who feel both self-hatred and the belief that others would be better off without them may engage in suicidal behaviour to relieve perceived suffering. This trait can develop over time in response to environmental and self-related factors [41] and may even be associated with unemployment, homelessness, and serious physical conditions, all of which possibly elevate vulnerability to suicidal behaviour. It should be noted that cognitive biases, low self-esteem, self-blame, shame, and physiological arousal further contribute to this construct [42,43]. As a multidimensional and dynamic phenomenon, perceived burdensomeness can change over time and in relationships with people, but perceptions of the outside world are formed by personality structure [44], with stronger personality structure reducing cognitive vulnerability [41]. In addition to perceived burdensomeness, hopelessness, particularly future-oriented negative thinking, was a key correlate of suicidal behaviour among women [42,45]. This finding suggests that interventions targeting maladaptive cognitive styles and fostering hope may be especially beneficial for this population.

In the men’s group, significant correlates of suicidal behaviours included an avoidance-focused coping style and the interaction between TB, PB, and capacity for suicide. Suicidal behaviours among men can be conceptualized as a maladaptive coping response to subjectively difficult situations. Men in this study demonstrated tendencies toward withdrawal and social alienation, consistent with lower help-seeking behaviours, which was expected in line with traditional masculinity norms. Suicidal acts in men appear to emerge from cognitive, emotional, and motivational failures to cope whereby escape becomes the only perceived solution. Masculine norms around the expression of anger and self-reliance may amplify this.

The interaction observed in men confirms key assumptions underlying IPTS. The combination of self-hatred, a desire to improve other people’s situation, a sense of alienation and loneliness, absence of support, lack of fear of dying, and an increased tolerance for physical pain appears necessary for an intentional suicide attempt. Specifically, thwarted belongingness becomes a significant correlate only when perceived burdensomeness and capacity for suicide are high. This pattern suggests that men’s suicidal behaviour is influenced by interpersonal deficits and a capacity to enact lethal self-harm, compounded by cognitive and emotional stressors (e.g., negative thoughts, emotions, and high levels of helplessness).

The study has several limitations. First, the criteria used to define the clinical group are constrained by the lack of reliable and valid diagnostic criteria concerning suicide independent of comorbid mental disorders [46,47,48]. DSM-5 and DSM-5-TR classify suicidal behaviour disorder as a disorder requiring further analysis, making it challenging to standardize diagnosis or determine approaches when mental disorders are absent [1,48,49]. This means that results cannot be generalized to clinical samples of individuals with diagnosed mental disorders. Most past research on gender differences in suicidal behaviours involves participants with diagnosed mental disorders, which limits comparability to the present sample [50]. Moreover, suicidal ideation was neither a required inclusion criterion nor included in the analyses, and its potential association with hopelessness constitutes a general limitation of the study. Additionally, intragroup sociodemographic differences were not analyzed.

A further limitation relates to the recruitment strategy. The clinical group consisted of individuals who had attempted suicide and were recruited from a single hospital in Poland, representing a very specific and hard-to-reach population. By definition, such a clinical population is likely to differ from the general population in terms of demographic and psychosocial characteristics, including employment status. Consequently, recruitment of a fully comparable control group is inherently difficult, and statistical matching (e.g., propensity scoring) was not feasible due to the unique and heterogeneous nature of the clinical group. These differences should be considered when interpreting the generalizability of our findings, particularly in the Polish context.

A further limitation concerns the relatively small sample size, which resulted in an EPV of approximately 5 in gender-stratified logistic regressions. Although below the conventional threshold of 10 [51], the study is exploratory and aims to identify personality predictors of suicidal behaviour based on the Interpersonal-Psychological Theory of Suicide and other personality variables. Results should therefore be interpreted with caution, and future studies with larger samples are warranted to confirm these findings [52,53].

In addition to the issues discussed above, focusing exclusively on neuroticism may have narrowed the conceptual scope of the model, as other personality traits could potentially account for additional variance in suicide-related outcomes. This raises the possibility of omitted variable bias and warrants caution in the interpretation of the findings.

The decision to limit the analysis to a single personality dimension was guided by both theoretical and empirical considerations. Neuroticism consistently demonstrates stronger and more robust associations with suicidality compared to other Big Five traits [34,35]. Methodological constraints related to sample size and statistical power further supported this choice, as including all five traits would have increased model complexity and reduced analytical power. Accordingly, neuroticism was selected as the most empirically supported and theoretically central personality trait within the context of the present study.

## 5. Conclusions

Despite its limitations, this study highlights gender-specific pathways of suicidal behaviour and has implications for targeted interventions. Identifying gender differences in factors associated with suicide is important for implementing specific actions aimed at reducing psychological distress, decreasing suicidal intent, preventing rehospitalization, and reducing suicidal behaviours. In the women’s group, suicidal behaviour appeared to be associated with cognitive–affective processes. Negative thinking about oneself and the world, both in the present moment and when considering the future, was associated with suicidal behaviours. These cognitive patterns may reflect internalized sense of entrapment and a perceived inability to affect change. Suicidal behaviour may therefore serve as an attempt to alleviate psychological pain and to restore a sense of relief or agency. The absence of a positive vision for the future intensifies feelings of powerlessness and hopelessness, increasing vulnerability to suicidal action. Interventions targeting maladaptive cognitive schemas, emotional regulation, and future-oriented thinking may therefore be especially beneficial. Moreover, gender-sensitive approaches that address sociocultural expectations and promote more equitable gender roles may help mitigate these risk pathways.

For the men’s group, suicidal behaviours appeared to emerge primarily from difficulties in coping with experiences such as loneliness, lack of perceived support, and self-hatred. Within this framework, suicidal behaviour may represent a maladaptive coping response to psychological distress, aligning with theoretical models that highlight the interaction of perceived burdensomeness, thwarted belongingness, and acquired capability. Because men often seek therapeutic help less frequently, these coping failures may go undetected, making suicide prevention in this group challenging. Approaches that prioritize adaptive coping, build self-esteem, and encourage help-seeking behaviours for emotion regulation and self-efficacy can be valuable.

Overall, the findings suggest that gendered expressions of suicidality are shaped by distinct psychosocial and cognitive–affective processes. Within the framework of the IPTS, these results indicate that women’s suicidality may be more closely linked to internalized cognitive distortions and affective entrapment, while men’s may reflect maladaptive coping arising from social disconnection and self-directed hostility. Tailoring prevention and intervention efforts to these differentiated pathways can enhance their effectiveness. Psychoeducation and cognitive bias modification may be helpful [54,55]. From a public mental health perspective, recognizing these gendered patterns offers insight for developing responsive and equitable suicide prevention strategies across clinical and community contexts.

## Figures and Tables

**Table 1:** Classification criteria for the study group (people with suicidal behaviour disorders)—authors’ own compilation based on DSM-5 and the literature.

Criterion	Description
Hospitalization	Hospitalization due to a suicide attempt (time criterion: suicide attempt in the last 24 months)
Time	24 months and less
Suicidal ideations	Suicidal ideations may have been present, but not required
Intentionality	Declaration that the intention behind the suicide attempt was death and not manipulative, unintentional behaviour; the behaviour was not undertaken for political purposes or as a religious manifesto
Nature of the self-destructive behaviour	The behaviour does not meet criteria for non-suicidal self-injury (NSSI) (DSM-5)
Criteria for exclusion	Respondent not qualifying when displaying Features/symptoms of active psychotic production; Symptoms/diagnosis of major depression; Symptoms associated with withdrawal from, or consumption of, psychoactive substances (abstinence syndrome, abstinence syndrome with delirium, or amnestic syndrome); Disorders caused by brain damage or dysfunction, mainly classified as F00–F09; Cognitive impairments; Features or diagnosis of disorders classified as F44.0–F44.9; Features or diagnosis of disorders classified as F70–F79

**Table 2:** Logistic regression model with interaction factors in the group of women.

	Statistics for the Block							
Variables Entered in the Blocks	χ^2^	*p*	Nagelkerke’s R^2^	B	SE	Wald(1)	*p*	Exp(B)
BLOCK 1	3.72	0.881	0.438					
avoidance-focused style				−0.012	0.031	0.142	0.707	0.988
hopelessness				0.243	0.095	6.548	0.011	1.276
neuroticism				0.051	0.034	2.268	0.132	1.052
BLOCK 2	9.46	0.305	0.466					
avoidance-focused style				−0.021	0.032	0.424	0.515	0.979
hopelessness				0.345	0.122	8.023	0.005	1.412
neuroticism				0.096	0.049	3.818	0.051	1.101
hopefulness				0.038	0.036	1.154	0.283	1.039
self-esteem				0.050	0.065	0.582	0.446	1.051
BLOCK 3	8.18	0.416	0.566					
avoidance-focused style				−0.026	0.035	0.538	0.463	0.975
hopelessness				0.288	0.147	3.846	0.050	1.334
neuroticism				0.105	0.055	3.657	0.056	1.110
overall result				0.031	0.045	0.481	0.488	1.032
self-esteem				0.121	0.082	2.178	0.140	1.129
perceived burdensomeness				0.114	0.053	4.631	0.031	1.121
thwarted belongingness				0.017	0.052	0.104	0.747	1.017
general capacity for suicide				0.033	0.039	0.697	0.404	1.033
BLOCK 4	8.23	0.412	0.571					
avoidance-focused style				−0.025	0.035	0.517	0.472	0.975
hopelessness				0.301	0.150	4.019	0.045	1.351
neuroticism				0.101	0.054	3.447	0.063	1.106
overall result				0.030	0.048	0.392	0.531	1.030
self-esteem				0.117	0.082	2.030	0.154	1.124
perceived burdensomeness				0.110	0.053	4.253	0.039	1.117
thwarted belongingness				0.026	0.054	0.229	0.632	1.026
general capacity for suicide				0.029	0.041	0.494	0.482	1.029
stand: perceived burdensomeness by stand: thwarted belongingness				0.475	0.692	0.471	0.492	1.608
BLOCK 5	8.08	0.426	0.578					
avoidance-focused style				−0.027	0.036	0.580	0.446	0.973
hopelessness				0.309	0.153	4.076	0.044	1.362
neuroticism				0.102	0.055	3.466	0.063	1.107
overall result				0.028	0.048	0.355	0.551	1.029
self-esteem				0.116	0.083	1.975	0.160	1.123
perceived burdensomeness				0.110	0.054	4.234	0.040	1.117
thwarted belongingness				0.039	0.056	0.481	0.488	1.039
general capacity for suicide				0.042	0.043	0.936	0.333	1.043
stand: perceived burdensomeness by stand: thwarted belongingness				0.703	0.669	1.106	0.293	2.020
stand: general capacity for suicide by stand: perceived burdensomeness by stand: thwarted belongingness				−0.493	0.470	1.102	0.294	0.611
BLOCK 6	8.57	0.380	0.581					
avoidance-focused style				−0.026	0.036	0.515	0.473	0.975
hopelessness				0.318	0.153	4.316	0.038	1.374
neuroticism				0.103	0.055	3.579	0.059	1.109
overall result				0.037	0.049	0.552	0.458	1.037
self-esteem				0.110	0.082	1.774	0.183	1.116
perceived burdensomeness				0.112	0.053	4.560	0.033	1.119
thwarted belongingness				0.021	0.060	0.122	0.727	1.021
general capacity for suicide				0.045	0.044	1.059	0.303	1.046
stand: perceived burdensomeness by stand: thwarted belongingness				0.670	0.683	0.962	0.327	1.954
stand: general capacity for suicide by stand: perceived burdensomeness by stand: thwarted belongingness				−0.717	0.620	1.338	0.247	0.488
stand: hopelessness by stand: perceived burdensomeness by stand: thwarted belongingness				0.433	0.664	0.426	0.514	1.543
constant				−13.548	5.083	7.105	0.008	0.000

**Table 3:** Logistic regression model with interaction factors in the group of men.

	Statistics for the Block in the Model							
Variables Entered in the Blocks	χ^2^	*p*	Nagelkerke’s R^2^	B	SE	Wald(1)	*p*	Exp(B)
BLOCK 1	5.14	0.743	0.398					
avoidance-focused style				0.075	0.031	5.794	0.016	1.078
hopelessness				0.043	0.112	0.146	0.703	1.044
neuroticism				0.109	0.043	6.472	0.011	1.115
BLOCK 2	9.98	0.266	0.482					
avoidance-focused style				0.112	0.040	7.667	0.006	1.118
hopelessness				−0.082	0.128	0.415	0.520	0.921
neuroticism				0.048	0.054	0.797	0.372	1.050
hopefulness				0.015	0.035	0.196	0.658	1.015
self-esteem				−0.157	0.064	6.143	0.013	0.854
BLOCK 3	11.77	0.162	0.611					
avoidance-focused style				0.129	0.051	6.344	0.012	1.137
hopelessness				−0.018	0.157	0.013	0.908	0.982
neuroticism				−0.017	0.068	0.064	0.800	0.983
overall result				0.043	0.045	0.923	0.337	1.044
self-esteem				−0.078	0.082	0.900	0.343	0.925
perceived burdensomeness				0.194	0.078	6.273	0.012	1.215
thwarted belongingness				−0.002	0.063	0.001	0.973	0.998
general capacity for suicide				−0.020	0.031	0.403	0.526	0.980
BLOCK 4	7.93	0.440	0.617					
avoidance-focused style				0.141	0.055	6.616	0.010	1.151
hopelessness				−0.005	0.156	0.001	0.975	0.995
neuroticism				−0.013	0.069	0.038	0.846	0.987
overall result				0.047	0.046	1.051	0.305	1.048
self-esteem				−0.087	0.081	1.143	0.285	0.917
perceived burdensomeness				0.167	0.071	5.520	0.019	1.181
thwarted belongingness				0.006	0.063	0.008	0.928	1.006
general capacity for suicide				−0.024	0.031	0.581	0.446	0.976
stand: perceived burdensomeness by stand: thwarted belongingness				0.495	0.612	0.655	0.418	1.641
BLOCK 5	11.70	0.165	0.649					
avoidance-focused style				0.162	0.062	6.903	0.009	1.176
hopelessness				−0.016	0.150	0.011	0.916	0.984
neuroticism				−0.021	0.074	0.080	0.777	0.979
overall result				0.082	0.052	2.471	0.116	1.086
self-esteem				−0.128	0.091	1.954	0.162	0.880
perceived burdensomeness				0.141	0.074	3.658	0.056	1.152
thwarted belongingness				0.016	0.072	0.049	0.825	1.016
general capacity for suicide				−0.101	0.055	3.431	0.064	0.904
stand: perceived burdensomeness by stand: thwarted belongingness				0.199	0.723	0.076	0.783	1.220
stand: general capacity for suicide by stand: perceived burdensomeness by stand: thwarted belongingness				1.690	0.851	3.943	0.047	5.421
BLOCK 6	11.74	0.163	0.650					
avoidance-focused style				0.162	0.062	6.831	0.009	1.176
hopelessness				−0.010	0.152	0.004	0.950	0.990
neuroticism				−0.023	0.075	0.094	0.759	0.977
overall result				0.079	0.053	2.196	0.138	1.082
self-esteem				−0.123	0.093	1.754	0.185	0.884
perceived burdensomeness				0.151	0.082	3.429	0.064	1.163
thwarted belongingness				0.026	0.079	0.108	0.742	1.026
general capacity for suicide				−0.100	0.054	3.475	0.062	0.905
stand: perceived burdensomeness by stand: thwarted belongingness				0.283	0.764	0.137	0.711	1.327
stand: general capacity for suicide by stand: perceived burdensomeness by stand: thwarted belongingness				1.665	0.823	4.091	0.043	5.286
stand: hopelessness by stand: perceived burdensomeness by stand: thwarted belongingness				−0.279	0.851	0.108	0.743	0.756
constant				−6.010	5.485	1.201	0.273	0.002

**Table 4:** Numbers in the individual groups formed on the basis of the interacting variables.

**Interaction of Variables**					
**Perceived Burdensomeness (PB) x General Capacity for Suicide (GCS)**		**Group No.**		**Frequency**	**Percentage**
	Important	1	PB = 0 AND GCS = 0	25	31.3
		2	PB = 0 AND GCS = 1	13	16.3
		3	PB = 1 AND GCS = 0	15	18.8
		4	PB = 1 AND GCS = 1	27	33.8
	Total			80	100.0
**Thwarted Belongingness (TB) x General Capacity for Suicide (GCS)**		**Group No.**		**Frequency**	**Percentage**
	Important	1	TB = 0 AND GCS = 0	28	35.0
		2	TB = 1 AND GCS = 1	12	15.0
		3	TB = 1 AND GCS = 0	12	15.0
		4	TB = 1 AND GCS = 1	28	35.0
	Total			80	100

**Table 5:** Thwarted belongingness and perceived burdensomeness as predictors of suicidal behaviours in the individual groups according to the values of other interaction variables.

Group	Predictor	Nagelkerke’s R^2^	B	SD	Wald	df	*p*	Exp(B)	95% CI for EXP(B)	
									LL	UL
1 PB x GCS	Thwarted belongingness	0.025	0.055	0.078	0.501	1	0.479	1.057	0.907	1.231
2 PB x GCS	Thwarted belongingness	0.039	0.049	0.078	0.385	1	0.535	1.050	0.900	1.224
3 PB x GCS	Thwarted belongingness	0.420	−0.140	0.075	3.484	1	0.062	0.870	0.751	1.007
4 PB x GCS	Thwarted belongingness	0.333	0.157	0.077	4.138	1	0.042	1.170	1.006	1.360
1 TB x GCS	Perceived burdensomeness	0.402	0.486	0.264	3.389	1	0.066	1.626	0.969	2.727
2 TB x GCS	Perceived burdensomeness	0.276	0.124	0.106	1.363	1	0.243	1.131	0.920	1.392
3 TB x GCS	Perceived burdensomeness	0.156	0.125	0.086	2.113	1	0.146	1.133	0.957	1.341
4 TB x GCS	Perceived burdensomeness	0.279	0.168	0.086	3.821	1	0.051	1.183	1.000	1.400

## Data Availability

The original data presented in the study are openly available in Open Science Framework at Anna Lubas-Grzyb and the Magdalena Rode project titled “Suicide”.
